# Efficacy of Adjunctive Chlorhexidine in non-surgical treatment of Peri-Implantitis/Peri-Implant Mucositis: An updated systematic review and meta-analysis

**DOI:** 10.12669/pjms.39.2.7253

**Published:** 2023

**Authors:** Mingfu Ye, Wenjun Liu, Shaolong Cheng, Lihui Yan

**Affiliations:** 1 Mingfu Ye, Department of Oral Implantology, Stomatological Hospital of Xiamen Medical College, Xiamen Key Laboratory of Stomatological Disease Diagnosis and Treatment, Xiamen 361008, Fujian Province, P.R. China; 2 Wenjun Liu, Department of Oral Implantology, Stomatological Hospital of Xiamen Medical College, Xiamen Key Laboratory of Stomatological Disease Diagnosis and Treatment, Xiamen 361008, Fujian Province, P.R. China; 3 Shaolong Cheng, Department of Oral Implantology, Stomatological Hospital of Xiamen Medical College, Xiamen Key Laboratory of Stomatological Disease Diagnosis and Treatment, Xiamen 361008, Fujian Province, P.R. China; 4 Lihui Yan, Department of Oral Implantology, Stomatological Hospital of Xiamen Medical College, Xiamen Key Laboratory of Stomatological Disease Diagnosis and Treatment, Xiamen 361008, Fujian Province, P.R. China

**Keywords:** Peri-implant disease, anti-microbial, dental implants, oral prophylaxis, Chlorhexidine

## Abstract

**Objective:**

The current review aimed to assess the efficacy of adjunctive chlorhexidine (CHX) in the non-surgical treatment of peri-implantitis/peri-implant mucositis.

**Methods:**

PubMed, Embase, Science Direct, CENTRAL, and Google Scholar databases were searched up to 10^th^ March 2022 for relevant randomized controlled trials or controlled clinical trials.

**Results:**

Fourteen studies were included. Meta-analysis revealed significantly lower probing depths in peri-implant mucositis patients using CHX adjuncts as compared to controls (SMD: -1.49 95% CI: -2.56, -0.42 I^2^=95% p=0.006). However, the same effect was not noted in peri-implantitis (SMD: -1.18 95% CI: -0.04, 2.40 I^2^=96% p=0.06). CHX was not found to improve bleeding of probing in peri-implant mucositis while sufficient data was unavailable for peri-implantitis. Results on other outcome variables were conflicting.

**Conclusion:**

Evidence on the efficacy of adjunctive CHX for peri-implant mucositis is conflicting. Similarly, strong conclusions on the effect of CHX for peri-implantitis cannot be drawn due to limited number of studies. Overall, there seems to be a trend of non-significant impact of CHX on outcomes of peri-implant mucositis as well as peri-implantitis.

## INTRODUCTION

Dental implants have become the primary mode of prosthetic rehabilitation of partially or completely edentulous patients. Indeed, trends from the USA suggest that there has been a 1000% increase in the use of dental implants from 1999 to 2016 and these numbers are bound to increase even further.^1^ The clinical course of dental implants is not without complications. Estimates suggest that around 19-65% and 1-47% of implants are affected by peri-implant mucositis and peri-implantitis, respectively.^2^ Peri-implant mucositis is a reversible inflammatory lesion affecting the mucosa surrounding an endosseous implant without loss of supporting peri-implant bone.^3^ Untreated peri-implant mucositis may lead to peri-implantitis which is clinically diagnosed by evidence of progressive marginal bone loss, probing depths of ≥6mm, and presence of bleeding on probing (BOP).^4^ Plaque is the most important initiator of peri-implant mucositis.^5^

Research also suggests that the anaerobic gram-negative bacterial flora seen in peri-implantitis is analogous to periodontitis.^6^ However, the clinical course of the disease may be modified based on several risk factors like prior history or concurrent presence of periodontitis, smoking, diabetes, prosthetic flaws, keratinized mucosa width, and lack of regular follow-up.^7^ Also, soft tissues surrounding an implant demonstrate significantly severe inflammatory reaction on exposure to oral biofilm and require a prolonged healing phase after biofilm removal when compared to soft-tissues surrounding natural teeth.^8^

The primary mode of treatment of peri-implant diseases consists of mechanical debridement. Surgical therapy may be utilized for peri-implantitis, however, the treatment has not been popular.^9^ Adjunctive therapies like chlorhexidine (CHX), minocycline, sodium hypochlorite, herbal mouthwashes, probiotics, air polishing, laser therapy, photodynamic therapy, and systemic antibiotics are also used.^10^ Of these, CHX is an easy-to-use topical antimicrobial that helps in the control and prevention of biofilm formation due to its high substantivity, bactericidal activity, and a broad spectrum of action.^11^

Recently, Liu et al^12^ in a systematic review assessed evidence on adjunctive CHX with non-surgical treatment of peri-implant disease but with small number of studies. With publication of several new studies, there is a need for updated evidence. Hence, this updated review aimed to answer the following clinical question: Does adjunctive topical CHX improve outcomes of peri-implant mucositis or peri-implantitis treated by non-surgical therapy?

## METHODS

The PRISMA statement^13^ (Preferred Reporting Items for Systematic Reviews and Meta-analyses) and recommendations of the Cochrane Handbook for Systematic Reviews of Intervention^14^ were followed. The PROSPERO registration no of the review was CRD42022315308.

### Literature search:

Two reviewers (M.Y. & W.L) conducted an electronic search of PubMed, Embase, Science Direct, CENTRAL, and Google Scholar databases up to 10^th^ March 2022. Any non-English language studies were translated to English using Google Translate. The search terms “chlorhexidine”, “peri-implantitis”, “peri-implant mucositis”, “dental implant”, “anti-microbial”, “anti-infective”, and “non-surgical” were used for all databases (Supplementary Table-I). Following the database search, we deduplicated the results. All the remaining studies were analyzed by their titles and abstracts. Articles relevant to the subject of our review were identified and their full texts were extracted. These articles were then examined for final inclusion in the review. The entire process was conducted by two reviewers (M.Y. & W.L). Any discrepancies in study selection were resolved by consensus.

**Supplementary Table-I:** Search details of PubMed database

Search number	Query	Search Details
1	(Chlorhexidine) AND (peri implantitis)	(“chlorhexidine”[MeSH Terms] OR “chlorhexidine”[All Fields] OR “chlorhexidin”[All Fields]) AND (“peri implantitis"[MeSH Terms] OR “peri implantitis"[All Fields] OR (“peri”[All Fields] AND “implantitis”[All Fields]) OR “peri implantitis"[All Fields])
2	(Chlorhexidine) AND (dental implant)	(“chlorhexidine”[MeSH Terms] OR “chlorhexidine”[All Fields] OR “chlorhexidin”[All Fields]) AND (“dental implants"[MeSH Terms] OR (“dental”[All Fields] AND “implants”[All Fields]) OR “dental implants"[All Fields] OR (“dental”[All Fields] AND “implant”[All Fields]) OR “dental implant"[All Fields])
3	(Chlorhexidine) AND (peri implant mucositis)	(“chlorhexidine”[MeSH Terms] OR “chlorhexidine”[All Fields] OR “chlorhexidin”[All Fields]) AND (“peri”[All Fields] AND (“embryo implantation"[MeSH Terms] OR (“embryo”[All Fields] AND “implantation”[All Fields]) OR “embryo implantation"[All Fields] OR “implantation”[All Fields] OR “implant”[All Fields] OR “implant s"[All Fields] OR “implantability”[All Fields] OR “implantable”[All Fields] OR “implantables”[All Fields] OR “implantate”[All Fields] OR “implantated”[All Fields] OR “implantates”[All Fields] OR “implantations”[All Fields] OR “implanted”[All Fields] OR “implanter”[All Fields] OR “implanters”[All Fields] OR “implanting”[All Fields] OR “implantion”[All Fields] OR “implantitis”[All Fields] OR “implants”[All Fields]) AND (“mucosalization”[All Fields] OR “mucosalized”[All Fields] OR “mucosally”[All Fields] OR “mucose”[All Fields] OR “mucoses”[All Fields] OR “mucositis”[MeSH Terms] OR “mucositis”[All Fields] OR “mucositides”[All Fields] OR “mucous membrane"[MeSH Terms] OR (“mucous”[All Fields] AND “membrane”[All Fields]) OR “mucous membrane"[All Fields] OR “mucosal”[All Fields]))
4	(anti-microbial) AND (peri implantitis)	"anti-microbial"[All Fields] AND (“peri implantitis"[MeSH Terms] OR “peri implantitis"[All Fields] OR (“peri”[All Fields] AND “implantitis”[All Fields]) OR “peri implantitis"[All Fields])
5	(anti-microbial) AND (dental implant)	"anti-microbial"[All Fields] AND (“dental implants"[MeSH Terms] OR (“dental”[All Fields] AND “implants”[All Fields]) OR “dental implants"[All Fields] OR (“dental”[All Fields] AND “implant”[All Fields]) OR “dental implant"[All Fields])

*Search number*	*Query*	*Search Details*

6	(anti-infective) AND (peri implantitis)	(“anti-infective agents"[Pharmacological Action] OR “anti-infective agents"[MeSH Terms] OR (“anti-infective"[All Fields] AND “agents”[All Fields]) OR “anti-infective agents"[All Fields] OR (“anti”[All Fields] AND “infective”[All Fields]) OR “anti-infective"[All Fields]) AND (“peri implantitis"[MeSH Terms] OR “peri implantitis"[All Fields] OR (“peri”[All Fields] AND “implantitis”[All Fields]) OR “peri implantitis"[All Fields])
7	(anti-infective) AND (peri implant mucositis)	(“anti infective agents"[Pharmacological Action] OR “anti infective agents"[MeSH Terms] OR (“anti infective"[All Fields] AND “agents”[All Fields]) OR “anti infective agents"[All Fields] OR (“anti”[All Fields] AND “infective”[All Fields]) OR “anti infective"[All Fields]) AND (“peri”[All Fields] AND (“embryo implantation"[MeSH Terms] OR (“embryo”[All Fields] AND “implantation”[All Fields]) OR “embryo implantation"[All Fields] OR “implantation”[All Fields] OR “implant”[All Fields] OR “implant s"[All Fields] OR “implantability”[All Fields] OR “implantable”[All Fields] OR “implantables”[All Fields] OR “implantate”[All Fields] OR “implantated”[All Fields] OR “implantates”[All Fields] OR “implantations”[All Fields] OR “implanted”[All Fields] OR “implanter”[All Fields] OR “implanters”[All Fields] OR “implanting”[All Fields] OR “implantion”[All Fields] OR “implantitis”[All Fields] OR “implants”[All Fields]) AND (“mucosalization”[All Fields] OR “mucosalized”[All Fields] OR “mucosally”[All Fields] OR “mucose”[All Fields] OR “mucoses”[All Fields] OR “mucositis”[MeSH Terms] OR “mucositis”[All Fields] OR “mucositides”[All Fields] OR “mucous membrane"[MeSH Terms] OR (“mucous”[All Fields] AND “membrane”[All Fields]) OR “mucous membrane"[All Fields] OR “mucosal”[All Fields]))
8	(non-surgical) AND (peri implantitis)	"non-surgical"[All Fields] AND (“peri implantitis"[MeSH Terms] OR “peri implantitis"[All Fields] OR (“peri”[All Fields] AND “implantitis”[All Fields]) OR “peri implantitis"[All Fields])
9	(non-surgical) AND (peri implant mucositis)	"non-surgical"[All Fields] AND (“peri”[All Fields] AND (“embryo implantation"[MeSH Terms] OR (“embryo”[All Fields] AND “implantation”[All Fields]) OR “embryo implantation"[All Fields] OR “implantation”[All Fields] OR “implant”[All Fields] OR “implant s"[All Fields] OR “implantability”[All Fields] OR “implantable”[All Fields] OR “implantables”[All Fields] OR “implantate”[All Fields] OR “implantated”[All Fields] OR “implantates”[All Fields] OR “implantations”[All Fields] OR “implanted”[All Fields] OR “implanter”[All Fields] OR “implanters”[All Fields] OR “implanting”[All Fields] OR “implantion”[All Fields] OR “implantitis”[All Fields] OR “implants”[All Fields]) AND (“mucosalization”[All Fields] OR “mucosalized”[All Fields] OR “mucosally”[All Fields] OR “mucose”[All Fields] OR “mucoses”[All Fields] OR “mucositis”[MeSH Terms] OR “mucositis”[All Fields] OR “mucositides”[All Fields] OR “mucous membrane"[MeSH Terms] OR (“mucous”[All Fields] AND “membrane”[All Fields]) OR “mucous membrane"[All Fields] OR “mucosal”[All Fields]))

### Eligibility criteria:

We formulated the inclusion criteria based on PICOS (Population, Intervention, Comparison, Outcome, and Study design). Studies with the following criteria were eligible:

1. *Population*: Adult patients (>18 years) with peri-implant mucositis or peri-implantitis
2. *Intervention*: Using any form of topical CHX for treating peri-implant mucositis or peri-implantitis with mechanical debridement
3. *Comparison*: Mechanical debridement without CHX or use of placebo
4. *Outcomes*: Reporting at least probing depth, BOP, and/or clinical attachment levels (CAL)
5. *Study design*: Randomised controlled trials (RCTs) or controlled clinical trials (CCTs)

All retrospective studies and in-vitro studies were excluded. We also excluded studies on zirconia implants, studies comparing CHX with any active treatment, studies combining CHX with surgical treatments, and those not reporting any of the relevant outcomes.

### Data extraction and quality assessment:

Data extracted included the first author, publication year, study location, study type, study population, CHX protocol, control group protocol, sample size, demographic details, study results, and follow-up. The primary outcomes of the review were probing depth, BOP, and CAL. We pooled data for these outcomes only if sufficient information was available from at least three studies. A descriptive analysis was conducted for all other outcomes.

We used the Cochrane Collaboration risk assessment tool for RCTs to assess the risk of bias.^15^ Studies were rated as low risk, high risk, or unclear risk of bias for: random sequence generation, allocation concealment, blinding of participants and personnel, blinding of outcome assessment, incomplete outcome data, selective reporting, and other biases.

### Statistical analysis:

The meta-analysis was performed using “Review Manager” (RevMan, version 5.3; Nordic Cochrane Centre (Cochrane Collaboration), Copenhagen, Denmark; 2014). A random-effects model was used for the analysis. We used standardized Mean Difference (SMD) with a 95% confidence interval (CI) to pool continuous data. Since some studies on peri-implantitis reported an only change of baseline scores, outcomes for peri-implantitis were pooled using such scores. A sensitivity analysis was also carried out. Heterogeneity was assessed using the I^2^ statistic. Since <10 studies were available for each analysis, funnel plots were not used to assess publication bias.

## RESULTS

**Fig.1:**
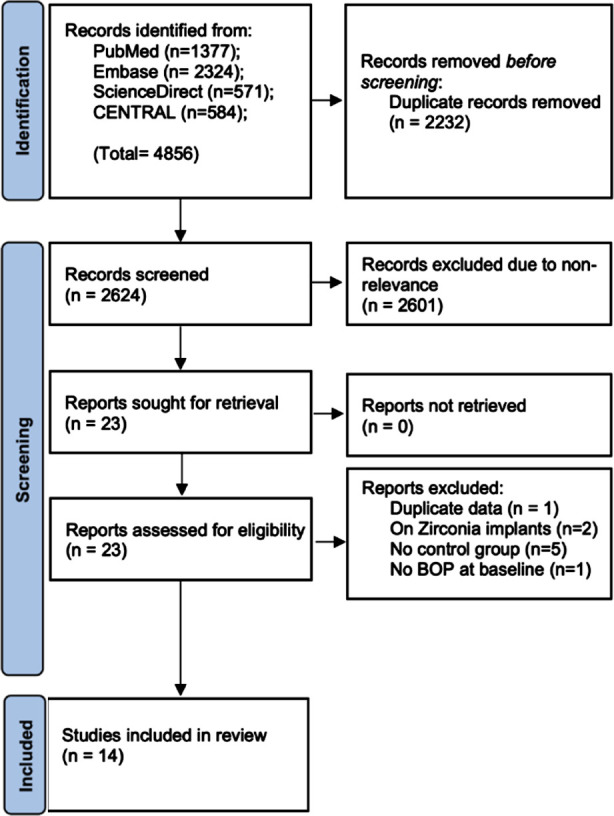
Study flow-chart

The search resulted in the identification of 4,782 unique articles (Fig.1). The authors selected 23 articles for full-text analysis. Of these, five were excluded with reasons and finally, a total of fourteen studies were included in the review.^3,16,17-28^ Except for two studies which were CCTs, all remaining studies were RCTs (Table-I). Four trials were on peri-implantitis while the rest were on peri-implant mucositis. Five studies used CHX only in mouthwash form, two studies used CHX chips, two used CHX irrigation, one used CHX gel to fill the peri-implant pocket, one used CHX gel for brushing, one used CHX irrigation and mouthwash, one used CHX gel along with mouthwash and tonsillar spray, while one used CHX irrigation, mouthwash, and gel application. There was significant heterogeneity in the CHX protocol amongst the included studies. The sample size of the CHX group ranged from 14 to 176 patients while that of the control group ranged from 12 to 174 patients.

**Table-I:** Characteristics of included studies

Author/Year	Location	Type	Study patients	CHX protocol	CON protocol	Groups	Sample size	Mean age (years)	Males (%)	Results	Follow-up
Porras 2002^20^	USA	RCT	Peri-implant mucositis	Mechanical cleansing with rubber cups and polishing paste; local irrigation of CHX; topical application of CHX gel and 0.12% CHX mouthwash BD for 10 days.	Same but without CHX	CHX CON	16 12	NR	NR	Reduction of plaque and inflammation, improvement in PD, gain in CAL seen with both CHX and CON groups. ddition of CHX did not enhance results.	1 and 3 months
Thone-Muhling 2010^17^	Germany	RCT	Peri-implant mucositis	Mechanical cleansing with plastic scalers and polyetheretherketone-coated ultrasonic instruments; once topical CHX gel application and CHX disinfection of tongue and tonsils; 0.2% CHX mouth rinse BD and tonsil spraying OD for 14 days.	Same but without CHX	CHX CON	22 14	46.3 53.3	66.7 80	Reduction in PD and BOP sites in both groups. Addition of CHX did not enhance results.	1, 2, 4, and 8 months
Heitz-Mayfield 2010^3^	Multi-national	RCT	Peri-implant mucositis	Mechanical debridement with titanium coated Gracey and carbon fiber curettes; brushing around the implant using CHX gel BD for 4 weeks.	Same with placebo gel	CHX CON	14 15	57 53	57.1 40	Reduction in number of BOP sites and PD in both groups. Addition of CHX did not enhance results.	1 and 3 months
Machtei 2012^21^	Israel	RCT	Peri-implantitis with PD of 6-10mm	Mechanical debridement with ultrasonic instruments; placement of up to four 2.5mg CHX chips; patients re-assessed at 2,4,6,8,12,18 weeks and chips re-inserted if PD >6mm.	Same with placebo chips	CHX CON	40 37	57.4 60.9	33.3 50	No significant difference in gain in CAL and reduction of PD between the two groups, reduction in number of BOP sites equal in both groups.	Up to 6 months

***Author/Year***	***Location***	***Type***	***Study patients***	***CHX protocol***	***CON protocol***	***Groups***	***Sample size***	***Mean age (years)***	***Males (%)***	***Results***	***Follow-up***

Levin 2015^16^	Israel	RCT	Peri-implantitis with PD of ≥5mm	Oral prophylaxis; use of water jet device containing 5ml CHX at home BD.	Same without water jet	CHX CON	19 20	NR	NR	No significant difference in reduction of PD and sites with BOP between CHX and CON groups	3 months
Menezes 2016^19^	Brazil	RCT	Peri-implant mucositis	Scaling and root planning; subgingival irrigation with 0.12% CHX three times within 10min; 0.12% CHX mouthwash BD 30mins after brushing for 14 days.	Same with placebo mouthwash	CHX CON	61 58	NR	21.3 8.6	Significant reduction of PD, BOP, GBI, PI in both groups. Addition of CHX did not enhance results.	1, 3 and 6 months
Crespi 2019^18^	Italy	CCT	Peri-implantitis with PD of ≥5mm	Mechanical debridement of implant surface with round bur without removal of granulation tissue; filling of peri-implant pocket by 0.2% CHX gel and 3% chlortetracycline hydrochloride gel around implant surface.	Same without gel placement; saline irrigation of pockets carried out for 1 min	CHX CON	40 35	64.2 63.5	33.3 45	Greater treatment success in study group. Significantly greater reduction of PD in CHX group.	3, 24, and 36 months
Alzoman 2020^22^	Pakistan	RCT	Peri-implant mucositis	Mechanical debridement; 10ml of 0.12% CHX mouthwash BD for 10 days	Same with placebo mouthwash	CHX CON	16 16	41.4 41.1	62.5 56.3	Significantly better reduction of BOP and PI with CHX as compared to CON	3, 6, and 12 weeks
Bunk 2020^23^	Germany	RCT	Peri-implant mucositis	Oral hygiene instructions; sub- and supramucosal mechanical debridement with titanium-curettes; polishing with rubber cup and low abrasive polishing paste; self-oral Irrigation with 50ml of 0.06% HCX solution OD.	Same with water irrigation	CHX CON	20 20	70 68.5	50 50	Better reduction of the presence and severity of peri-implant mucositis with CHX.	4, 8, and 12 weeks
Philip 2020^25^	Netherlands	RCT	Peri-implant mucositis	Mechanical debridement with ultrasonic device and high-tech plastic material coated tip; 0.2% CHX mouthwash BD.	Same with placebo mouthwash	CHX CON	30 28	62 65	53.3 57.1	No significant difference in BOP and PI between CHX and CON groups at final follow-up	1 and 3 months
Ahmedbeyli 2021^26^	Azerbaijan	CCT	Peri-implant mucositis	Mechanical debridement with air abrasive device, Gracey’s curette, and individualized oral hygiene training; 0.05% CHX mouthwash BD for 10 days.	Same but without CHX	CHX CON	15 16	NR	NR	Significantly better reduction of BOP and PD with CHX as compared to CON	2 weeks, 1, 3 and 6 months
Bollain 2021^24^	Spain	RCT	Peri-implant mucositis	Mechanical debridement with ultrasonic device using plastic tip; air-polishing with erythritol; 0.03% CHX and 0.05% cetylpyridinium chloride mouthwash BD.	Same with placebo mouthwash	CHX CON	27 27	61.4 61	59.3 48.1	No significant difference in BOP, PD and PI between CHX and CON groups	6 and 12 months
Machtei 2021^27^	Multi-national	RCT	Peri-implantitis with PD of 5-8mm	Mechanical debridement at baseline and bi-weekly; repeated CHX chips (up to two chips/pocket	Mechanical debridement only	CHX CON	176 174	62.5 62.6	37.7 43.8	Significantly better reduction of PD with CHX	8, 12, 16, 24 weeks
Alqutub 2022^28^	Saudi Arabia	RCT	Peri-implant mucositis	Mechanical debridement; 0.12% CHX mouthwash BD for 2 weeks	Same with water mouthwash	CHX CON	15 15	52.1 51.2	60 53.3	Significantly better reduction in PD, PI and gingival index with CHX	12 weeks

### Probing Depth:

**Fig.2:**
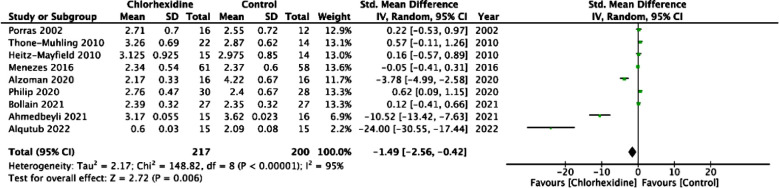
Forrest plot of probing depth for peri-implant mucositis

Meta-analysis revealed significantly lower probing depths in patients using CHX adjuncts as compared to controls (SMD: -1.49 95% CI: -2.56, -0.42 I^2^=95% p=0.006) (Fig.2). On sequential exclusion of three studies, there was no statistically significant difference between CHX and control groups. These studies were: Alqutub et al^28^ (SMD: -0.82 95% CI: -1.17, 0.07 I^2^=93% p=0.07); Ahmedbeyli et al^26^ (SMD: -0.63 95% CI: -1.55, 0.28 I^2^=93% p=0.17); and Alzoman et al^22^ (SMD: -0.98 95% CI: -2.00, 0.03 I^2^=94% p=0.06).

**Fig.3:**
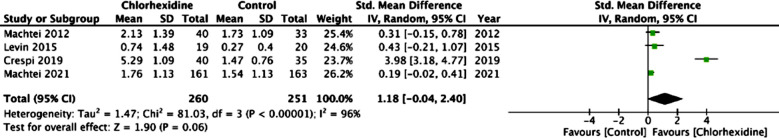
Forrest plot of probing depth for peri-implantitis

Meta-analysis revealed no significant difference in change in probing depths between CHX and control groups (SMD: -1.18 95% CI: -0.04, 2.40 I^2^=96% p=0.06) (Fig.3). On the exclusion of the study of Crespi et al^18^, the results revealed a significantly greater change in probing depths with CHX as compared to control (SMD: 0.23 95% CI: 0.05, 0.42 I^2^=0% p=0.01).

### BOP:

**Fig.4:**
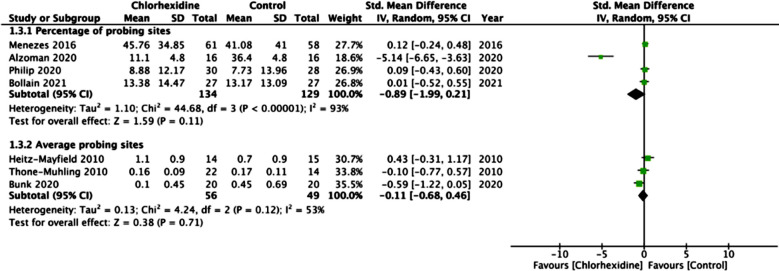
Forrest plot of BOP for peri-implant mucositis

Seven studies reported BOP as a percentage of probing sites while three reported data as an average of probing sites. On meta-analysis of studies reporting data as a percentage of probing sites, we noted no statistically significant difference between CHX and control groups (SMD: -0.89 95% CI: -1.99, 0.21 I^2^=93% p=0.11).(Fig.4) Similarly, no difference was noted between CHX and control groups on a pooled analysis of studies reporting data as an average of probing sites (SMD: -0.11 95% CI: -0.68, 0.46 I^2^=53% p=0.71) Fig-4. These results were stable on sensitivity analysis. Sufficient data was not available for meta-analysis of the BOP for peri-implantitis.

### Other outcomes:

Details of other outcomes reported by the studies are presented in Supplementary Table-II. For peri-implant mucositis, Alqutub et al^28^ noted a significantly better reduction in modified plaque index and modified gingival index with CHX as compared to control. Similarly, a better reduction of plaque index was noted by Alzoman et al^22^ with CHX. Ahmedbeyli et al^26^ reported significantly better improvement of the gingival bleeding index while Porras et al^20^ noted significantly higher CAL with CHX as compared to controls.

**Supplementary Table-II:** Descriptive analysis of other outcomes reported by the included studies.

Study	Outcome	Results
***Peri-implant mucositis***		
Porras 2002^20^	BOP	No significant difference between study and control groups
	Modified sulcus bleeding index	No significant difference between study and control groups at any time points.
	CAL	Significantly higher change in study group as compared to control group
	Plaque scores	No significant difference between study and control groups at any time points.
Thone-Muhling 2010^17^	Plaque index	No significant difference between study and control groups
	Gingival index	No significant difference between study and control groups
Heitz-Mayfield 2010^3^	Mean total DNA count	No significant difference between study and control groups
Menezes 2016^19^	Visible plaque index	No significant difference between study and control groups
	Gingival bleeding index	No significant difference between study and control groups
Alzoman 2020^22^	Plaque index	Significantly lower scores in study group as compared to control group
Bunk 2020^23^	Mucositis severity score	No significant difference between study and control groups
	Modified plaque index	No significant difference between study and control groups
Philip 2020^25^	Modified bleeding index	No significant difference between study and control groups
	Modified plaque index	No significant difference between study and control groups
Ahmedbeyli 2021^26^	Gingival bleeding index	Significantly lower scores in study group as compared to control group
	Simplified oral hygiene index	No significant difference between study and control groups
Bollain 2021^24^	Plaque index	No significant difference between study and control groups
Alqutub 2022^28^	Modified plaque index	Significantly lower scores in study group as compared to control group
	Modified gingival index	Significantly lower scores in study group as compared to control group
***Peri-implantitis***		
Machtei 2012^21^	BOP	No significant difference between study and control groups
	CAL	No significant difference between study and control groups
Levin 2015^16^	BOP	Significantly higher reduction of BOP sites in study group as compared to control group
Crespi 201918	BOP	Significantly higher reduction of BOP sites in study group as compared to control group
	CAL	Significantly better improvement in study group as compared to control group
	Mucosal recession	Significantly better improvement in study group as compared to control group
	Marginal bone levels	Significantly better improvement in study group as compared to control group

BOP, bleeding on probing; CAL, clinical attachment level.

For studies on peri-implantitis, Machtei et al^21^ reported no difference in BOP and CAL with and without the use of CHX. However, Crespi et al^18^ and Levin et al^16^ reported significantly better improvement in outcomes with CHX as compared to controls.

### Risk of bias analysis:

**Supplementary Fig.1:**
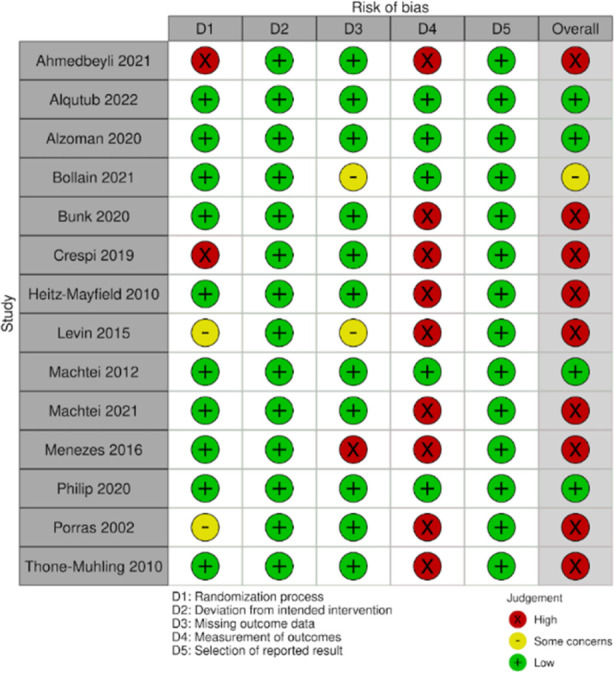
Risk of bias summary. Green, low risk of bias; Yellow, unclear risk of bias; Red, high risk of bias.

Risk of bias in the included studies as per author’s judgement are presented in Supplementary Fig.1.

## DISCUSSION

The cause of both gingivitis and peri-implant mucositis has been attributed to the pathological effects of oral biofilm.^29^ Indeed, while the host response to biofilm does not differ much between teeth and implants, BOP is more frequently observed around implants as compared to teeth.^30^ Also, peri-implantitis presents with larger lesions along with a significantly higher destructive inflammatory profile and faster clinical progression.^31^ Probing depths are higher with implants and there is a tendency of the probe to reach the alveolar bone relatively easily as compared to teeth. While biofilm reduction is an effective treatment for both types of diseases, root surfaces are easier to access and clean in teeth as compared to implants due to design features and surface roughness of the latter.^30^ This has led to the use of several adjuncts to manage peri-implant diseases.^10^

Barootchi et al^32^ in a recent review including 14 RCTs concluded that adjunctive therapies had no significant impact on clinical outcomes as compared to non-surgical therapy alone. While the authors assessed the efficacy of CHX, only six RCTs could be included. Another meta-analysis by Ramanauskaite et al^33^ assessed the efficacy of numerous adjunctive therapies with non-surgical therapy of peri-implant diseases only to conclude that adjunctive measures provided no beneficial effect in resolving peri-implant mucositis. Similar to the previous review, the studies on CHX were limited. On the other hand, Liu et al^12^ reviewed only on adjunctive use of CHX and concluded that it has no beneficial effect with non-surgical management of peri-implant diseases. However, it analyzed just seven studies. Therefore, our review represents a major update from the previous study^12^, by including seven more studies.

In case of peri-implant mucositis, our meta-analysis revealed statistically significant reduction in probing depth with the use of CHX adjunct. This is in contrast with the results of Liu et al^12^ who noted no difference in probing depths with CHX but with only four trials. The difference in our results could be due to the inclusion of five more trials. However, our results were not stable on sensitivity analysis. On sequential exclusion of three studies the results turned statistically non-significant. Also, on the forest plot, it can be noted that the studies of Alqutub et al^28^ and Ahmedbeyli et al^26^ were outliners reporting a large difference between the study and control groups. The cause of such large difference in these two studies is difficult to contemplate as both trials used CHX mouthwashes for just 10-14 days.

Similar protocol was used by other trials but without any difference in probing depths between CHX and control groups. Also, in our analysis, we noted no difference in BOP in peri-implant mucositis patients with and without adjunctive CHX. In the overall analysis, only one study of Alzoman et al^22^ reported a significant reduction of BOP sites with the use of CHX mouthwash. None of the remaining studies noted any difference between the study and control groups. Also, on descriptive analysis of other outcomes, most did not differ between the study and control groups.

A limited number of trials have examined the efficacy of CHX for peri-implantitis as only four studies were available. We noted that adjunctive use of CHX did not significantly impact probing depths in patients with peri-implantitis. However, it is important to note that the 95% CI were wide ranging from -0.04 to 2.40, with the lower end very close to zero, indicating a greater change of probing depths with CHX. On examination of the forest plot it can be seen that the study of Crespi et al^18^ reported significantly better outcomes with CHX as compared to other trials. This variation may be explained by the difference in the method of CHX application between the trials. Crespi et al^18^ used a combination gel of 3% chlortetracycline hydrochloride and CHX which was placed around the implant surface, while the other trials used CHX irrigation or only CHX chips.

Use of the gel may have reduced the bacterial load and detoxified the implant surface in their study resulting in better outcomes. The authors also left the granulation tissue in the soft tissue pocket hypothesizing that it would result in proliferation of cells with embryonic stem cell properties thereby leading to better healing of tissues.^34^ This may be the reason that Crespi et al^18^ noted statistically significant improvement in all outcomes in the CHX group as compared to the control group.

There was significant heterogeneity in all our meta-analyses. This was expected and is in line with previous reviews^12^ due to the different CHX protocols used by the included studies. The authors used CHX in chips, mouthwashes, gels, irrigating solutions, sprays and even in combinations. Furthermore, there was no homogeneity in the timing and duration of CHX use. The difference in patient populations, severity of illness, and other implant-related factors could have also led to this substantial inter-study heterogeneity. Future studies should standardize the CHX protocol and also compare different forms of CHX to generate quality evidence.

Overall, our results do not clearly prove the added efficacy of CHX for peri-implant diseases. Such lack of effect of CHX may be due to the variation in substantivity of the drug between tooth and implant surfaces. In contrast to tooth surface wherein CHX has high substantivity with long-lasting effect, the adhesion of CHX on implant surfaces depends on surface texture and the drug concentration.^35^ Research indicates that adsorbed CHX is rapidly released of non-treated implant surfaces, while prepared implant surfaces (sand blasting/acid etching) may have better CHX uptake.^36^ There are also concerns regarding the alteration of implant surfaces by anti-microbial agents. Kotsakis et al^37^ have noted that CHX can affect the biocompatibility of implant surface and recommend against the use of CHX on implant surface.

### Limitations:

Firstly, most of the studies were of small sample size and could have been underpowered to detect significant differences. Secondly, as discussed earlier, there was vast heterogeneity in the method and timing of CHX application. Thirdly, the studies also varied in the type of outcomes reported which resulted in lower number of studies in the meta-analysis. Lastly, the number of studies on peri-implantitis were too few to derive strong conclusions.

### Strength of the study:

The strength and uniqueness of the review is that it is the largest meta-analysis till date assessing the efficacy of adjunctive CHX for non-surgical treatment of peri-implantitis/peri-implant mucositis. A comprehensive detailed literature search was conducted wherein we doubled the number of studies from the previous review^12^. We believe that by combining data from published studies this review provides high quality evidence to clinicians involved in the management of peri-implant diseases. The results of this review will allow informed decisions and provide impetus to further research on CHX. Based on the results of the study, at this point it is unclear if CHX should be routinely used as an adjunct to managing peri-implant diseases. However, due to conflicting results, it is advised that clinicians may evaluate each case on its merit and recommend the usage of CHX till further data is available.

## CONCLUSION

Evidence on the efficacy of adjunctive CHX for peri-implant mucositis is conflicting. Similarly, strong conclusions on the effect of CHX for peri-implantitis cannot be drawn due to limited number of studies. Overall, there seems to be a trend of non-significant impact of CHX on outcomes of peri-implant mucositis as well as peri-implantitis. Further research is needed assessing the efficacy of specific delivery of CHX on outcomes of peri-implant diseases.

### Authors’ contributions:

**MY:** conceived and designed the study.

**WL, SC and LY:** collected the data and performed the analysis.

**MY**: was involved in the writing of the manuscript and is responsible for the integrity of the study.

All authors have read and approved the final manuscript.
